# Application of WGM Resonances to the Measurement of the Temperature Increment of Ho and Ho-Yb Doped Optical Fibers Pumped at 1125 and 975 nm

**DOI:** 10.3390/s21062094

**Published:** 2021-03-17

**Authors:** Xavier Roselló-Mechó, Martina Delgado-Pinar, Yuri O. Barmenkov, Alexander V. Kir’yanov, Miguel V. Andrés

**Affiliations:** 1 Department of Applied Physics and Electromagnetism-ICMUV, University of Valencia, C/Dr. Moliner 50, 46100 Burjassot, Spain; miguel.andres@uv.es; 2 Photonics Department, Centro de Investigaciones en Óptica, Loma del Bosque 115, León 37150, Mexico; yuri@cio.mx (Y.O.B.); kiryanov@cio.mx (A.V.K.)

**Keywords:** whispering gallery modes, holmium, ytterbium, doped fibers, fiber characterization, 2 μm fiber lasers

## Abstract

Optical fiber characterization using whispering gallery mode resonances of the fiber itself has been demonstrated to be a powerful technique. In this work, we exploit the thermal sensitivity of whispering gallery mode resonances to characterize the pump-induced temperature increment in holmium doped and holmium-ytterbium codoped optical fibers. The technique relies on the measurement of the resonances’ wavelength shift due to temperature variation as a function of the pump power. Holmium doped fibers were pumped to the second excited level ${}^{5}$I${}_{6}$ of the Ho${}^{3+}$ ion using a laser diode at 1125 nm and ytterbium-holmium codoped fibers to the ${}^{2}$F${}_{5/2}$ level of the Yb${}^{3+}$ ion by a laser diode at 975 nm. Our results demonstrate that pumping ytterbium-holmium codoped fibers at 975 nm results in dramatic thermal effects, producing a temperature increment two orders higher than that observed in holmium doped fibers pumped with a 1125 nm laser diode.

## 1. Introduction

The excitation of the azimuthal whispering gallery mode (WGM) resonances has been used to characterize different parameters in spherical and cylindrical microresonators. The wavelength shift of WGM resonances has been used to measure diameter fluctuations along a fiber [1,2], Pockel’s coefficients [3], thermal effects in microspheres [4,5], erbium and ytterbium doped fibers [6], fiber Bragg gratings (FBGs) [7], and UV irradiated photosensitive fibers [8]. Some other techniques employed to measure temperature variations and thermal effects in fibers involve the use of FBGs [9], tapers [10,11], interferometric techniques [10,11,12], or Brillouin sensors [13]. The technique based in WGM is a non-invasive, non-destructive technique that allows characterizing special, pristine fibers without requiring any post-processing. The typical high Q-factor of the resonances (10${}^{6}$–10${}^{7}$ in silica cylinders) allows achieving low detection limits (0.06 ${}^{\circ }$C in our setup) and provides a large range of measurement without the ambiguity inherent to interferometric techniques. In addition, this technique provides an axial resolution below the mm level that allows measuring at localized points along a device, and it does not require long sections of fibers to perform the characterization.

Over the last two decades, advances in the performance of fiber lasers led to the adoption of this technology in several areas and applications in photonics, such as high power systems [14]. Most of the fiber lasers developed to date are based on silica fibers doped with three rare-earth elements: erbium (Er${}^{3+}$), ytterbium (Yb${}^{3+}$), and thulium (Tm${}^{3+}$) [15]. The widespread use of these three chemical elements among the many others that have been investigated is due to different factors. Some of the key features that make these elements relevant are the following: Ytterbium shows conversion efficiencies up to 50%, thus allowing its use for the manufacturing of high power fiber lasers. Erbium shows an emission band in the telecommunications wavelength range, while thulium’s emission band is centered at 2 μm. Each dopant, or their combination with ytterbium, is exploited to develop novel light sources located in these two wavelength ranges, for several applications such as telecommunication, materials’ processing, bio-imaging, etc. In turn, the applications of these specific doped fibers have driven the development of fiber-compatible components at the absorption and emission wavelengths, such as reliable pump diodes, isolators, fiber couplers, etc. In particular, it is worth highlighting that Yb and Er doped silica fibers show emission bands centered at ∼1 μm and ∼1.5 μm, respectively, which meet the requirements for a huge variety of applications: in continuous wave (CW) high power lasers, in telecommunication technologies, in operational configurations for the generation of short giant pulses, etc. [16]. On the other hand, the fibers doped with Tm${}^{3+}$ present a broadband gain at ∼2 μm, a spectral region with increasing applications, for example, in the development of light sources for use in medical surgery, eye-safe technology (e.g., LIDAR), or spectroscopy [17]. As an alternative to Tm${}^{3+}$, holmium (Ho${}^{3+}$) is an interesting option to develop fiber lasers centered in the spectral range above ∼2 μm [18,19]. The fibers doped with Ho${}^{3+}$ exhibit attractive properties for use in the design and fabrication of lasers: high efficiencies, a good power scaling, and an operational band spectrally located in a high transmission atmospheric window [20]. One of the main challenges with these types of fiber lasers is designing a proper pump system. The majority of reported holmium doped fiber lasers use an in-band pumped configuration by employing thulium doped fiber lasers [18] or a direct laser diode [21]. The wavelength of the pump source must be selected to match one of the two absorption bands of Ho${}^{3+}$, which are centered at ∼1.15 μm and at ∼1.95 μm [22,23]. In order to allow pumping at optical wavelengths outside the absorption bands of Ho${}^{3+}$, codoped Ho fibers have been developed for application in fiber lasers, such as reported in [24,25,26]. Some designs have emerged by codoping the holmium fibers with other rare-earth ions, such as Yb${}^{3+}$ [24]. These variants enable the employment of pump sources at wavelengths out of the absorption bands of Ho${}^{3+}$ [25].

As mentioned, one of the main applications of the fibers doped with Ho${}^{3+}$ is the development of high power lasers beyond 2 μm, both CW and pulsed, which require significantly large pump powers [27]. Due to the non-radiative transitions that may occur in the active medium, this leads to a temperature increment of the fibers when they are under the operational regime. The heating may affect the performance of the fiber laser, for example by shifting the spectral position of the fiber Bragg gratings (FBGs) included in the setup to provide the optical feedback in a Fabry–Pérot laser scheme and by changing the absorption and emission spectra of active fibers. Therefore, the study of the pump-induced heating suffered by doped fibers is a topic of interest [6].

In this work, we employ a technique based on the temperature sensitivity of the WGM resonances in order to characterize the temperature increment of doped fibers. In silica microresonators with diameters comparable to those of optical fibers, the optical field of the WGMs is mainly located inside the resonator, bounded to the outer surface; thus, they will be highly sensitive to variations in the physical properties of the microresonator material. Since these microresonators exhibit intrinsically low losses and the guiding mechanism is the total internal reflection, the WGM resonances show extremely high Q-factors [28], enabling the measurement of small variations of the microresonator parameters, with a low detection limit. Another important advantage of the technique is that the WGM resonances are excited at a given point on the fiber, which enables monitoring the variations along the fiber. In the case of a thermal characterization, the temperature profile can be measured with high precision [7]. We propose to exploit the WGM measurement technique to determine the influence of the pump-induced heating on different parameters such as the concentration of Ho${}^{3+}$, the introduction of Yb${}^{3+}$ as a codopant, and the use of two different pump wavelengths (975 nm and 1125 nm).

## 2. Theoretical Description

### 2.1. WGM Thermal Sensitivity

The spectral position of the WGM resonances is set by the refractive index of the resonator cladding material and its diameter. Both parameters depend on the temperature and lead to heating-induced shifts of the resonance. The relative wavelength shift, Δλ, of the WGM resonances as a function of a given temperature increment, ΔT, can be expressed as follows:

(1) $$\frac{\Delta \lambda }{\lambda }\;=\;\left(\frac{1}{R}\frac{dR}{dT}\;+\;\frac{1}{n_{\mathrm{eff}}}\frac{dn_{\mathrm{eff}}}{dT}\right)\Delta T,$$

where λ is the resonant wavelength of the unperturbed resonator, *R* the radius of the fiber, and $n_{\mathrm{eff}}$ the effective refractive index of the WGMs. This shift is ruled by the thermo-optic effect and the thermal expansion coefficient of the material. For cladding-silica fibers, like the ones treated in this work, the shift rate of the resonances was measured in previous works to be 8.2pm/${}^{\circ }$C, and it allows correlating the temperature increment with the WGM resonances’ shift [6]. Moreover, accurate numerical simulations determined that the different WGM resonances in the vicinity of 1.55 μm show the same shift rates with temperature, simplifying the utility of the technique since the modal labeling of the resonances is not necessary [29]. Finally, since the WGM resonances are excited at a given point of the microresonator, the use of these modes enables axial characterization of the thermal profile of the fiber under test with a spatial resolution limited by the axial extension of the WGMs, which for our devices is ∼200 μm [28].

### 2.2. Energy Level Diagram of the Yb/Ho Codoped Fibers

**Figure 1:**
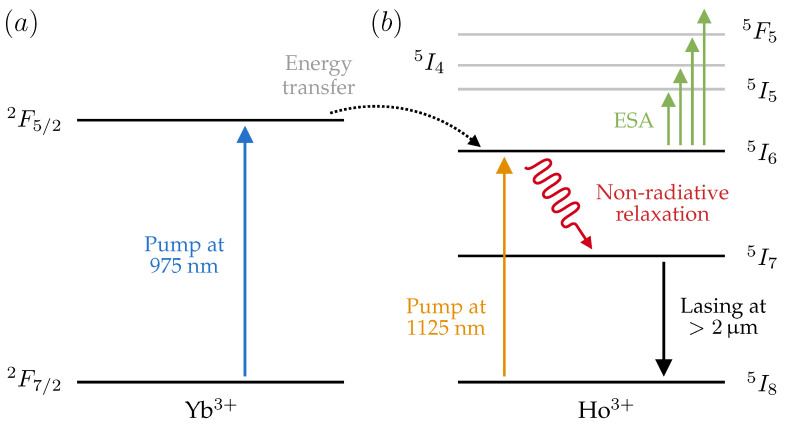
Simplified diagram of the energy levels of (**a**) Yb^3+^ and (**b**) Ho^3+^ ions, as well as the processes involved to generate lasing beyond 2 μm with pump signals at 975 and 1125 nm

For this work, we investigated the thermal effects in two Ho${}^{3+}$ doped fibers. Figure 1 presents the energy levels of Yb${}^{3+}$ (Figure 1a), Ho${}^{3+}$ (Figure 1b), and the energy transfer mechanism between both systems. The first fiber we investigated was an Ho${}^{3+}$ fiber without any other dopants. In this case, we exploited the ${}^{5}$I${}_{6}$ absorption band of Ho${}^{3+}$ to pump the fiber; this absorption band is centered at ∼1150 nm [30]. We employed an in-band configuration, by pumping at 1125nm. The second fiber included Yb${}^{3+}$ as a codopant, together with Ho${}^{3+}$. In this case, we pumped outside the absorption bands of Ho${}^{3+}$, at 975nm, by exploiting the ${}^{2}F_{5/2}$ band of ytterbium (see Figure 1a) and the consequent energy transfer mechanism to the ${}^{5}$I${}_{6}$ band of Ho${}^{3+}$ [26].

Figure 1 shows a simplified scheme of the energy levels of the ion multi-system Ho${}^{3+}$/Yb${}^{3+}$ and the processes involved to generate lasing at >2 μm. For a 975 nm pump wavelength, the Yb${}^{3+}$ ions in the ground state, ${}^{2}F_{7/2}$, are excited to the energy level ${}^{2}F_{5/2}$. Part of the energy is then transferred to the Ho${}^{3+}$ ions, resulting in the excitation to the ${}^{5}I_{6}$ energy level. Then, after a non-radiative multiphonon relaxation to the ${}^{5}I_{7}$ energy level, photons with a wavelength beyond 2 μm are emitted. The second option consists of using 1125 nm to pump the active medium, which results in a direct excitation of Ho${}^{3+}$ ions from the ground level ${}^{5}I_{8}$ to the second excited level ${}^{5}I_{6}$. Again, a non-radiative relaxation occurs in the decay to the ${}^{5}I_{7}$ (laser) level, which ends up lasing at 2 μm in the decay to the ground state. The complete description of the energy levels in the Yb/Ho system and the processes during the pump illumination can be found in detail in [26]. The non-radiative processes that are present in the Ho${}^{3+}$/Yb${}^{3+}$ subsystems are responsible for the temperature increment suffered by the doped fibers. It is important to note that the quantum efficiency of the radiative decay from the laser level is rather low (∼7–10%) due to partially multiphonon relaxation [31,32], which results in additional thermal loss.

## 3. Experimental Procedure

**Figure 2:**
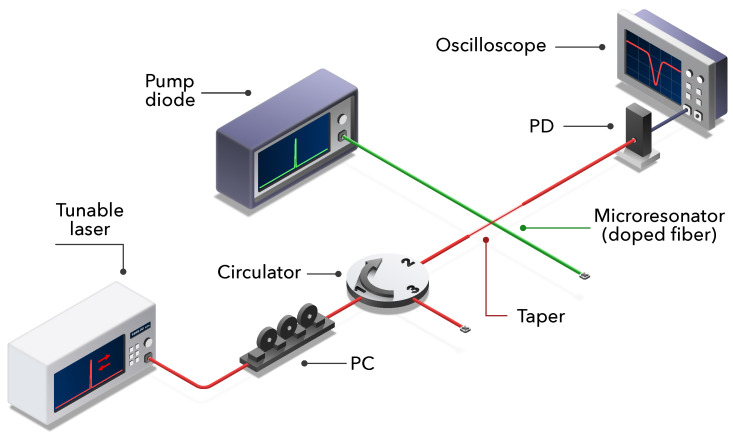
Scheme of the experimental setup employed to characterize the temperature increment by means of the whispering gallery modes (WGMs). PD, photodiode; PC, polarization controller.

**Figure 3:**
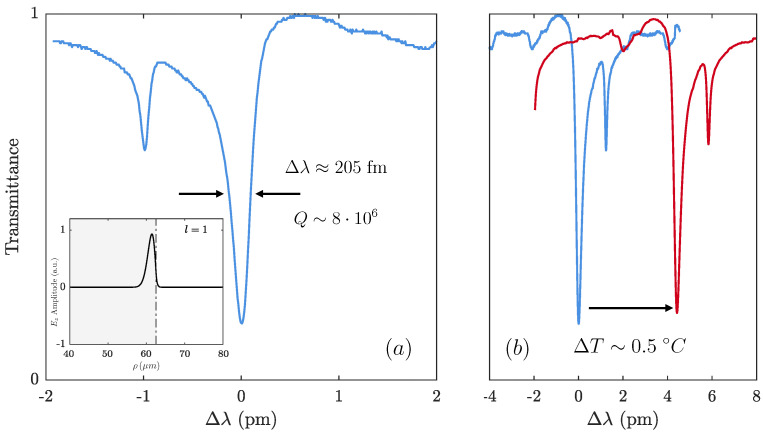
(**a**) Transmission trace of a typical WGM resonance (inset: radial profile of a fundamental mode of a WGM resonance (l = 1 and m = 354); (**b**) illustrative example of the wavelength shift suffered by the resonances for a temperature increment of ΔT = 0.5${}^{\circ }$C.

Figure 2 shows a scheme of the experimental setup employed both to pump the doped fibers and to characterize the response of the WGM resonances. To excite the WGM resonances in the doped fibers, which play the role of the microresonators, we used an auxiliary tapered silica fiber of a 2 μm waist and a 5mm length [33]. We tapered the diameter of a conventional optical fiber by employing a fuse and pull technique [34]. The taper and the microresonator were placed perpendicularly. The distance between taper and resonator was controlled by a 3 axis flexure stage; although, in order to prevent undesired noise in the measurements, both elements were placed in contact. Moreover, the taper was swept along the doped fibers to characterize their thermal profile. The observed resonances showed a typical 3 dB linewidth 204fm, corresponding to a Q-factor of $\left(7.61\;\pm \;0.05\right)\cdot 10^{6}$. Figure 3a,b shows an illustrative example of the wavelength shift suffered by the WGM resonances as the temperature of the optical fiber increases, to illustrate the operational principle of our example.

To excite the WGM resonances, we employed a tunable, linearly polarized laser (linewidth < 300kHz) with a tuning range that covers 1515 to 1545nm. By employing the piezoelectric tuning facility of the laser, we performed a continuous scanning of the emitted signal. A polarization controller (PC) was placed to optimize the excitation of one of the WGM polarizations, either TE (transversal electric) or TM (transversal magnetic). Since the thermo-optic effect is intrinsically isotropic, both polarizations respond identically to a given heating, that is a given value of the pump power. A circulator was placed before the tapered fiber to measure the WGM reflected signal as an alternative to the measurement of the notches in transmission. The transmitted signal of the taper was measured by means of a photodiode (PD), whose output electric signal was registered by an oscilloscope (OSC) synchronized with the signal that drives the wavelength shift of the laser. It is noteworthy that the optical field of the WGMs is strongly located in the cladding-air interface, mostly within the resonator, in a shell of about 3 μm in width for the fundamental radial order mode [35]. Since the dopants are in the core of the fiber, the WGM field distribution does not overlap with them, and then, it does not affect the excitation of the Ho${}^{3+}$ and Yb${}^{3+}$ ions. On the other hand, although the source of the heating is located in the core of the doped fiber, it distributes over the transversal section of the fiber with the result of a mostly uniform transversal temperature profile, where the difference of temperature between the core of the fiber and the vicinity of the cladding-air interface is smaller than 1.5% [36].

The pump signal launched through the doped fiber was provided by two pigtailed, single-mode, CW laser diode sources with a maximum power of 400mW operating at different wavelength: (i) at 1125nm and (ii) at 975nm. We characterized three different types of home-made doped fibers: two holmium doped fibers (HDFs) with different concentrations (doping level ratio of HDF2/HDF1∼1.5) and an ytterbium-holmium codoped fiber (YHDF). The absorption losses of HDF1 and HDF2 at 1125nm are ∼3.6 dB/m and ∼6.7 dB/m, respectively. For the YHDF, at 978nm, it presents ∼2100 dB/m, while at 1125nm, it is ∼7 dB/m, which is similar to the loss of HDF2. Sections of a few centimeters long of doped fibers were studied in this work. The fibers under test were spliced to a Fibercore SM980 and to the pigtailed pump diode. The temperature increment was measured at ∼1 mm after the splice. The detailed information about the fiber parameters, dopant concentrations, and the process of fabrication can be found in [24,30] for HDFs and the YHDF, respectively.

## 4. Results and Discussion

**Figure 4:**
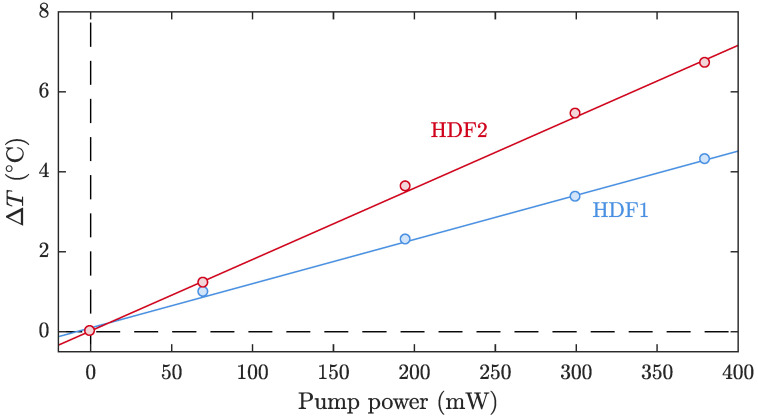
Measurement of the temperature increment of Holmium Doped Fiber 1 (HDF1) and HDF2 as a function of the pump signal power. Pump wavelength centered at 1125nm.

First, we measured the thermal shift of the WGM resonances excited in the HDFs at ∼1 mm after the splice, when the 1125nm pump signal was launched through them. Figure 4 shows the temperature increment in HDF1 and HDF2 as a function of the launched power. The heating rate of HDF1 was 11.37 ± 0.14${}^{\circ }$C/W, while the rate for HDF2 was 18.16 ± 0.18${}^{\circ }$C/W. HDF2 suffered a temperature increment ∼1.6 times larger than HDF1. This result is in concordance with the concentration ratio of the dopant used for the fabrication of the fibers, which is HDF2/HDF1∼1.5. As can be observed in Figure 4, in the power range studied, the heating showed a linear trend with the pump power, and there was no sign of saturation or nonlinearity. Exploiting the capability to measure the thermal profile of the fibers, we characterized the temperature increment at different positions along the HDFs. Along a length of 15mm from the splice between the HDF and the SMF980 fiber used for pump delivery, the heating rate showed the same linear trend, and no significant variations were observed. This allowed us to assume that the pump of the fibers was uniform along the sections of fiber under study, for this length of the resonators/fibers.

**Figure 5:**
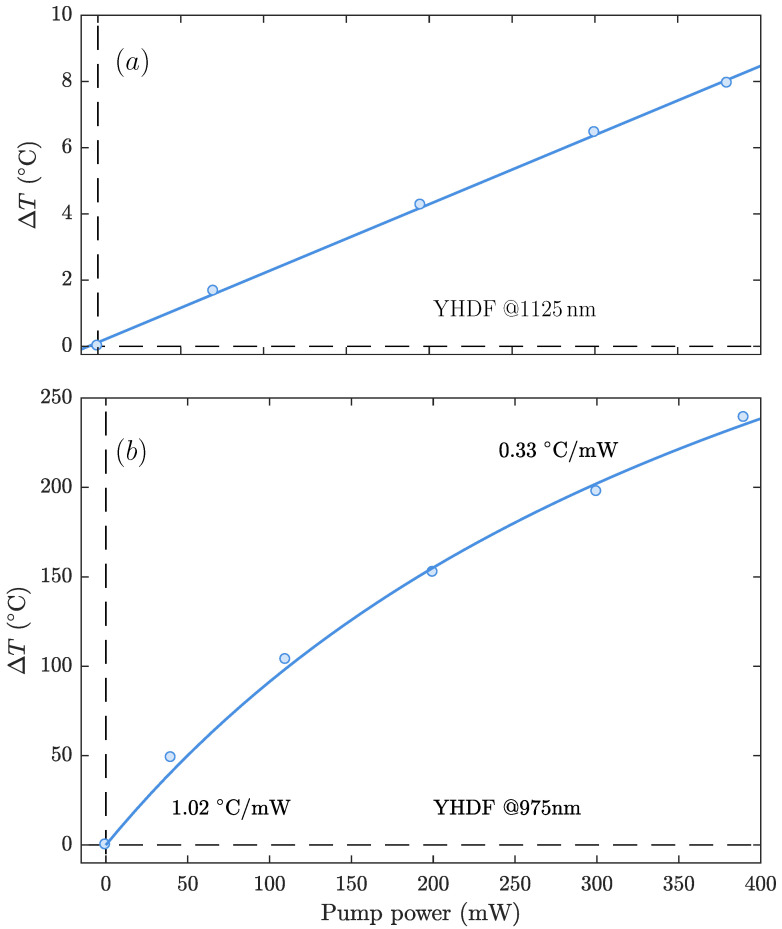
Temperature increment of the ytterbium-holmium codoped fiber (YHDF) as function of the pump signal at two different wavelengths: (**a**) at 1125nm; (**b**) at 975nm. Circles and curves show experimental points and fits, respectively.

Figure 5a shows the temperature increment of the YHDF as a function of the launched power at 1125nm. The heating rate was 21.4 ± 0.2${}^{\circ }$C/W, showing a linear trend with no evidence of saturation or nonlinearity in the studied power range. Since the concentration of Ho${}^{3+}$ in the YHDF is similar to that in HDF2, the temperature increments in these two fibers are comparable. Then, the same section of the YHDF was pumped at 975nm, exploiting the indirect excitation of the multi-system Ho/Yb. Figure 1 and Figure 5b show that the temperature increment experienced by the YHDF was much greater than for the previous scenario. At 400mW of pump power, the fiber reached a temperature increment that exceeded 200${}^{\circ }$C.

The heating strongly deviated from a linear response: the best fit was obtained using a standard formula for saturating process $\Delta T\;=\;kP/\left(P\;+\;P_{\mathrm{sat}}\right)$, where *k* is a coefficient, *P* is the pump power, and $P_{\mathrm{sat}}$ is the saturating pump power. The formula was obtained on the assumption that the main limiting factor for the temperature increase is saturation of the population of Yb${}^{3+}$ ions in the excited state, which limits energy transfer from Yb${}^{3+}$ ions to Ho${}^{3+}$ ions. By fitting the experimental data to this formula, the saturation pump power in the YHDF at 975 nm: $P_{\mathrm{sat}}\;=\;460\;$mW (value of the coefficient: k ≈ 514) can be obtained. It was also observed that the local heating rates decreased as the pump power increased. In particular, the heating rate around 50mW of pump power was three times the value around 300mW of pump power (the local slopes’ values of the curve are indicated next to the fitting curve in Figure 5b). For this fiber, we observed a significant dependence of the heating as a function of the axial position, as a result of the high absorption coefficient of the fiber at this wavelength. We measured the heating at different points along the fiber and concluded that the pump power was mostly absorbed after 5cm. Thus, we set the position for measurements shown in Figure 5b at 1 mm from the splice of the YHDF and a section of SM980 (the fiber used for pump delivery) and carefully repeated every characterization at the same position.

Therefore, when the YHDF is pumped with a signal at 975nm, the induced thermal effects can be critical. In other experiments involving Yb/Ho codoped fibers pumped around 1 μm, the authors employed a pump source at 1064nm where the absorption coefficient was dramatically smaller (∼2100 dB/m at 978nm and ∼3.4 dB/m at 1064nm) [24].

## 5. Conclusions

The WGM technique employed in this work allowed us to perform a thermal characterization of the pump-induced heating suffered by holmium doped and ytterbium-holmium codoped silica fibers. This technique enables the characterization of fibers with different concentrations and types of dopants and their response to pump signals at different wavelengths. The HDFs pumped at 1125nm showed a direct correlation between the heating and the concentration of dopant. On the other hand, the YHDF showed a critical temperature increment when pumped at 975nm, exceeding 200${}^{\circ }$C for 400mW of pump power. The information from the thermal characterization might be used in the design of new active Ho/Yb fibers, a key component in the manufacturing of all-fiber laser cavities emitting beyond 2 μm, in order to study different concentrations of rare-earth ions to enhance the radiative processes and reduce the pump-induced heating important for lasers’ optimization.

## Data Availability

Not applicable.
